# Multiscale Evaluation of Mechanical, Microstructural, and Chemical Properties of Weathered Aggregates on the Qinghai–Tibet Plateau

**DOI:** 10.3390/ma18163816

**Published:** 2025-08-14

**Authors:** Huijing Liu, Xin Li, Haisheng Ren, Xue Zhang, Yicheng Shuai, Xinhang Wu, Wu Bo

**Affiliations:** 1 School of Engineering, Xizang University, Lhasa 850000, China; 18089318626@163.com (H.L.); 18729173512@163.com (X.Z.); 17783218203@163.com (Y.S.); wxhabc2023@163.com (X.W.); 2 Plateau Major Infrastructure Smart Construction and Resilience Safety Technology Innovation Center, Lhasa 850000, China; 15708313017@163.com (X.L.); ren_hs510@seu.edu.cn (H.R.); 3 Tibet Tianlu Co., Ltd., Lhasa 850000, China; 4 Intelligent Transport System Research Center, Southeast University, Nanjing 211189, China

**Keywords:** weathered aggregates, multiscale characterization, pore connectivity, Si/Al ratio, gray entropy model, elemental migration, mechanical degradation, SEM–EDS analysis, Qinghai–Tibet Plateau

## Abstract

The Qinghai–Tibet Plateau presents a unique challenge for infrastructure development due to its extreme geological and climatic conditions—high elevation, large diurnal temperature fluctuations, frequent freeze–thaw cycles, intense ultraviolet radiation, and seasonal precipitation. These factors greatly accelerate the weathering of rock materials, leading to aggregates with increased porosity, microcracking, and weakened mechanical properties. While the engineering implications of such degradation are evident, the underlying material science of weathered aggregates—particularly their microstructure–property relationships—remains insufficiently explored, necessitating further investigation to inform material selection and design. In this study, three representative types of weathered aggregates (silica-rich, carbonaceous, and alumina-rich), alongside unweathered natural aggregates, were examined through both macro-scale (density, water absorption, crushing value, abrasion resistance) and micro-scale (scanning electron microscopy (SEM), energy-dispersive spectroscopy (EDS)) analyses. To capture the material evolution, we introduced a simplified classification framework based on the Si/Al ratio and porosity and applied a gray entropy correlation model to quantify the coupling between microstructure and mechanical performance. Results show that weathering reduces the Si/Al ratio from 2.45 to 1.82, increases porosity from 4.2% to 12.7%, enlarges the average pore size to 0.85 μm, raises microcrack density to 1.40 μm/μm^2^, and increases the proportion of connected pores to 68.2%. These microstructural degradations correlate with decreased aggregate density, increased water absorption (up to 8.0%), higher crushing value (27.4%), and abrasion resistance loss (26.0%). Based on these findings, a weathered aggregate classification and pretreatment strategy is proposed, offering a practical reference for engineers to improve material performance in high-altitude road construction.

## 1. Introduction

The Qinghai–Tibet Plateau, the highest and one of the most geologically complex regions on Earth, is subjected to extreme environmental stressors such as severe cold, low oxygen pressure, intense ultraviolet radiation, and frequent freeze–thaw cycles [1,2]. These factors accelerate bedrock weathering, resulting in aggregates with significantly altered mineralogical compositions and weakened microstructures [3,4,5]. The engineering application of such weathered aggregates in road construction has become a major challenge, especially with the rapid development of transportation infrastructure in plateau areas [6,7,8].

Weathered aggregates are generally characterized by low density, high porosity, and poor mechanical integrity, which severely affect their load-bearing performance and durability [9,10,11]. Traditional material specifications—developed primarily for plain or low-altitude environments—do not adequately address the unique deterioration mechanisms found in high-altitude weathered materials [12,13,14,15]. While numerous studies have investigated the physical degradation of aggregates, such as reduced crushing strength and increased water absorption, few have explored their microstructural evolution or established effective micro–macro correlation models [16,17,18,19].

Previous research has identified various weathering types in the plateau region, including silica-rich, carbonaceous, and alumina-rich aggregates, each representing distinct mineralogical origins and weathering paths [4,20,21,22]. However, a systematic classification framework that integrates microstructure and compositional indicators remains underdeveloped. Although SEM and EDS technologies have been used to observe pore morphology and elemental distribution, existing applications are often qualitative, lacking integration with performance-based evaluation [23,24,25].

To address these gaps, this study establishes a multiscale evaluation model for weathered aggregates, focusing on three representative types (silica-rich, carbonaceous, alumina-rich) sourced from the Qinghai–Tibet Plateau [26,27,28,29]. The key innovation lies in a dual-factor control framework combining the Si/Al ratio and porosity as performance-sensitive indicators. This framework enhances the classification accuracy compared to conventional schemes that rely solely on physical or mineralogical parameters [30,31,32,33,34,35].

Furthermore, a gray entropy correlation model is employed to analyze the coupling relationships between microstructural characteristics and macroscopic performance. Unlike conventional models such as PCA or multivariate regression, gray entropy is particularly suitable for small-sample and incomplete datasets typical of field-collected aggregates, offering greater robustness in complex environments [36,37,38,39,40,41].

In addition, the study investigates the elemental migration process during weathering and highlights the role of mineral transformations involving phases such as hematite and illite. These secondary minerals are inferred to contribute to strength loss and pore development, supported by mineralogical references and SEM–EDS observations [33,34,42,43,44].

In summary, this work presents three major contributions:

(1) A regional classification framework for weathered aggregates is proposed based on the integration of compositional indicators (e.g., Si/Al ratio) and microstructural features (e.g., porosity, microcracks). This approach moves beyond traditional methods that rely solely on geological origin or physical appearance, offering a more deterioration-sensitive and environment-specific classification strategy that reflects intrinsic degradation mechanisms in plateau aggregates;
(2) A quantitative multiparameter analysis framework is developed by combining SEM imaging, ImageJ (version 1.54f; National Institutes of Health, Bethesda, MD, USA) pore extraction, and EDS elemental mapping. This integration enables a simultaneous assessment of pore connectivity and elemental migration, providing an effective tool for identifying microstructural signatures responsible for performance degradation and freeze–thaw susceptibility;
(3) A gray entropy-based micro–macro coupling model is constructed and embedded within a dual-factor evaluation system using Si/Al ratio and porosity as predictive variables. Compared to conventional models such as PCA or multivariate regression, this approach demonstrates stronger adaptability to incomplete, nonlinear, and small-sample datasets, which are typical of aggregates from field environments in cold regions. The model supports adaptive classification, performance prediction, and material selection in high-altitude engineering contexts.

## 2. Materials and Methods

### 2.1. Materials

**Figure 1:**
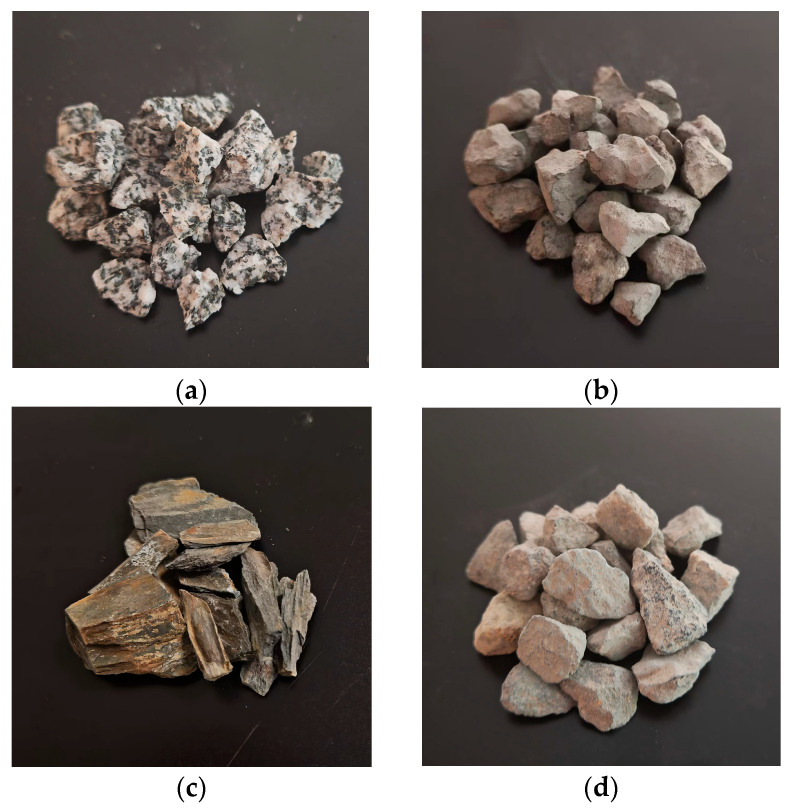
Representative macroscopic appearance of natural and weathered aggregates used in this study: (**a**) natural aggregate with compact texture and sharp edges; (**b**) silica-rich weathered aggregate showing surface roughness and granular disintegration; (**c**) carbonaceous weathered aggregate characterized by laminated and flaky morphology; (**d**) alumina-rich weathered aggregate with visibly softened edges and severe surface degradation.

**Figure 2:**
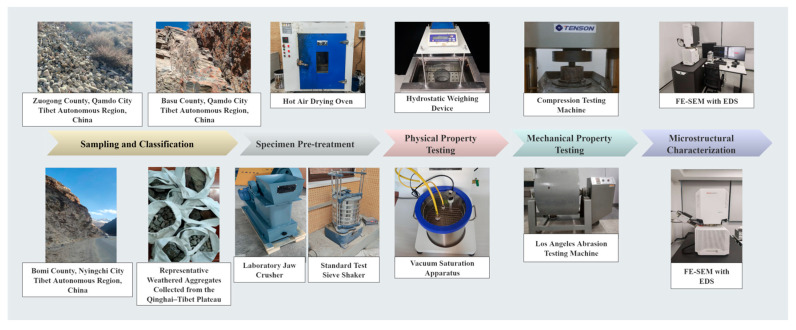
Experimental workflow of the multiscale analysis of weathered aggregates.

All aggregate materials used in this study were sourced locally along National Highway 318 in the eastern Qinghai–Tibet Plateau to ensure regional representativeness and engineering relevance. A total of 28 bulk samples were collected from three counties—Zuogong, Basu, and Bomi—each representing distinct geological and weathering conditions. The parent rock of all aggregates is weathered granite, a lithology typical of the plateau. Among the collected materials, four aggregate types were classified: one natural aggregate (minimal weathering) and three weathered types based on dominant elemental enrichment—silica-rich (Si > 24 wt.%), carbonaceous (C > 4.5 wt.%), and alumina-rich (Al > 11.5 wt.%). All coarse aggregates were crushed and sieved to the standard size range of 9.5–13.2 mm, and fine aggregates were screened to <4.75 mm. To ensure uniform initial conditions, all specimens were cleaned with distilled water and oven-dried at 110 ± 5 °C until constant mass. Aggregate classification was based on visual weathering features (e.g., surface color, cracking patterns) and elemental composition thresholds measured by EDS. Representative appearances of each aggregate type are presented in Figure 1. A complete workflow of the experimental program is provided in Figure 2, summarizing the sampling, classification, and multiscale characterization procedures.

### 2.2. Test Methods

#### 2.2.1. Bulk Density and Water Absorption

Bulk density and water absorption tests were performed in accordance with ASTM C127 (coarse aggregates) [45] and ASTM C128 (fine aggregates) [46]. Prior to testing, samples were washed with distilled water and oven-dried at 110 ± 5 °C until a constant mass was reached. Dried samples were immersed in water at room temperature—natural aggregates for 24 ± 2 h, and weathered aggregates for at least 48 ± 4 h due to higher porosity. After soaking, aggregates were surface-dried to a saturated surface-dry (ssd) condition. The ssd mass, oven-dry mass, and submerged mass were recorded. Each test was repeated three times per aggregate type, and mean values were reported. Water density was assumed as 1.00 g/cm^3^. The following equations were used:

(1) $$\mathrm{Bulk}\;\mathrm{Specific}\;\mathrm{Gravity}\;(\mathrm{ssd})\;=\frac{W_{ssd}}{W_{ssd}-W_{water}}$$

(2) $$\mathrm{Apparent}\;\mathrm{Specific}\;\mathrm{Gravity}=\frac{W_{dry}}{W_{dry}-W_{water}}$$

(3) $$\mathrm{Water}\;\mathrm{Absorption}\;(\%)=\frac{W_{ssd}-W_{dry}}{W_{dry}}\times 100$$

where $W_{dry}$ is the oven-dry mass of the sample; $W_{ssd}$ is the saturated surface-dry mass; $W_{water}$ is the submerged mass; and rater density is assumed to be 1.00 g/cm^3^. These measurements provide a quantitative evaluation of how weathering affects the internal pore structure and water uptake capacity of the aggregates, which are critical parameters for understanding subsequent mechanical behavior.

#### 2.2.2. Crushing Value Test

The crushing value test was conducted in accordance with the Chinese national standard GB/T 14685–2011 [47]. This test evaluates the resistance of aggregates to compressive loading [17,48]. Aggregates were dried and sieved to 9.5–13.2 mm. For each group, 3000 g of sample was placed in three layers in a steel cylinder, each layer compacted with 25 tamping blows. The sample was then loaded in a universal testing machine at a rate of 1 kN/s until reaching 400 kN, which was held for 5 s. After loading, the crushed material was sieved through a 2.36 mm mesh, and the mass of particles passing the sieve was recorded. Each aggregate type was tested three times, and the mean crushing value was calculated as

(4) $$CV\left(\%\right)=\frac{W_{passing}}{W_{total}}\times 100$$

where $W_{total}$ is the original mass of the sample before testing; $W_{passing}$ is the mass of the crushed material finer than 2.36 mm. A higher crushing value indicates lower resistance to compressive loads and therefore poorer aggregate integrity. During the test, weathered aggregates commonly exhibited granular disintegration and flaky peeling failure modes. These failure characteristics were documented simultaneously to provide macro-level verification for the underlying microstructural degradation mechanisms.

#### 2.2.3. Abrasion Value Test

The abrasion resistance of aggregates was evaluated using the Los Angeles (LA) abrasion test in accordance with JTG E42-2005 [49] (Specifications for Highway Engineering Aggregate Tests) [50,51]. This test simulates aggregate degradation under mechanical wear caused by traffic loading. For each trial, a 5000 ± 50 g aggregate sample was placed in a Los Angeles abrasion drum along with 12 standard steel spheres (diameter: 48 mm). The drum was rotated at 31 ± 2 rpm for 500 revolutions. After testing, the sample was sieved through a 1.7 mm mesh, and the mass of the fine material passing the sieve (M_pass) was recorded. Each aggregate type was tested three times, and the mean value was calculated using

(5) $$LAA\left(\%\right)=\frac{W_{fine}}{W_{initial}}\times 100$$

where $W_{initial}$ is the initial mass of the test sample; $W_{fine}$ is the mass of material passing the 1.7 mm sieve after testing. This index reflects the aggregate’s resistance to wear and mechanical degradation under cyclic loading. It is a critical parameter for designing asphalt mixtures, particularly in regions subject to extreme environmental and loading conditions, such as the Qinghai–Tibet Plateau.

#### 2.2.4. Pore Structure Characterization by SEM

**Figure 3:**
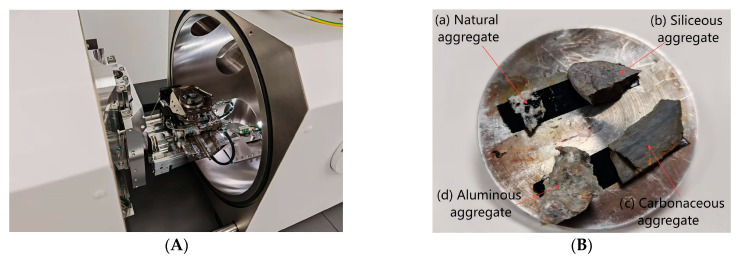
SEM observation setup and sample geometry. (**A**) SEM chamber with precision stage for high-resolution imaging of aggregate microstructures. (**B**) Mounted aggregate specimens: (**a**) natural aggregate; (**b**) silica-rich aggregate; (**c**) carbonaceous aggregate; (**d**) alumina-rich aggregate. All samples were cut to uniform size and fixed with conductive adhesive to ensure imaging stability and minimize charging.

To investigate the microstructural characteristics of weathered aggregates, scanning electron microscopy (SEM) was employed in conjunction with ImageJ image analysis software to quantitatively assess pore morphology and structural parameters for both natural and weathered aggregates (silica-rich, carbonaceous, and alumina-rich) [52,53]. Representative aggregate specimens were cut into blocks with dimensions of approximately 5 × 5 × 3 mm^3^. These were ultrasonically cleaned using acetone, followed by absolute ethanol to remove surface impurities. After cleaning, the samples were dried in a vacuum oven at 60 °C for 24 h to preserve their original pore structures. Figure 3 illustrates the preparation and imaging setup.

To improve imaging resolution and contrast, a conductive platinum coating was applied to the specimens: 5 nm for natural aggregates and 8 nm for weathered aggregates due to differences in surface conductivity. SEM imaging was conducted using a FEI Quanta 650 system (FEI Company, Hillsboro, OR, USA) under an accelerating voltage of 10–15 kV. Backscattered electron (BSE) mode was used to capture fracture surface images at magnifications ranging from ×1000 to ×50,000, ensuring complete pore structure visualization. Image processing was performed using ImageJ software. Grayscale threshold segmentation was applied to isolate pore regions, and pore area fraction (porosity) was calculated as follows:

(6) $$\mathrm{Porosity}\left(\%\right)=\frac{A_{\mathrm{pore}}}{A_{\mathrm{total}}}\times 100$$

where $A_{pore}$ is the total area of pores in the image; $A_{total}$ is the total image field area. Further analysis was conducted to quantify dissolution pits (aspect ratio > 2) and microcracks (width > 100 nm). A skeleton extraction algorithm was applied to evaluate the proportion of connected pores. Imaging was performed at regions approximately 200 μm below the aggregate surface. For each specimen type, three distinct fields of view were analyzed, and the average values were reported. The relative standard deviation for measured parameters was controlled within 5%. This method enables comprehensive quantification of key microstructural indicators such as porosity, average pore size, microcrack density, and pore connectivity. These data provide a critical foundation for interpreting macroscopic performance degradation. The testing protocol followed SY/T 5162-2014 [54], Characterization of Pore Structure in Rock by SEM (China).

#### 2.2.5. Elemental Migration Analysis by EDS

To investigate mineralogical changes and spatial elemental distribution during the weathering process, Energy-Dispersive X-ray Spectroscopy (EDS) was utilized to analyze the elemental migration behavior in the aggregates [34,55]. The analysis was performed in conjunction with SEM imaging to ensure precise targeting of characteristic microstructural regions. Testing focused on both primary mineral zones and weathered zones, such as crack boundaries, dissolution pits, and altered rims. For each selected area, a surface scan was conducted over a 50 × 50 μm^2^ field at a spatial resolution of 0.5 μm (i.e., step size), collecting elemental distribution data for Si, Al, Fe, Ca, Mg, K, and other major elements. In addition, line-scan analysis was performed along 10 μm-wide belts set perpendicular to dominant cracks. Spectral points were collected every 2 μm across each transect, with a dwell time of 30 s per point to ensure spectral stability and accuracy. Based on the EDS results, an elemental Migration Index (MI) was calculated to quantify migration intensity using the following formula:

(7) $$MI=\frac{C_{crack}-C_{matrix}}{C_{matrix}}$$

where $C_{crack}$ represents the atomic concentration of the element at the crack zone; $C_{matrix}$ represents the corresponding concentration in the unaltered matrix zone. Positive MI values indicate elemental enrichment near cracks, while negative values suggest elemental depletion. Comparative analysis of key elements such as Si, Al, and Fe, combined with SEM-derived pore structure images, helps elucidate weathering-induced elemental migration pathways and the formation of secondary minerals. To ensure data reliability, each analytical area was scanned at least twice, and point-to-point measurement errors were controlled within ±0.3 at.%. The entire testing procedure adhered to the national standard GB/T 17361-2013 [56], General Rules for Microbeam Analysis Techniques, ensuring methodological consistency and data comparability.

## 3. Results and Discussion

### 3.1. Density and Water Absorption

Table 1 summarizes the effects of weathering on aggregate density and water absorption. Weathering exerts a substantial impact on the density and water absorption characteristics of aggregates. Natural aggregates exhibited the most favorable performance, with a water absorption rate of only 1.0% and a saturated surface-dry (SSD) density of 2.65 g/cm^3^. In contrast, weathered aggregates showed a consistent trend of increasing water absorption and decreasing density. Among them, the alumina-rich aggregate exhibited the most severe degradation, with a water absorption rate of 8.0% and an SSD density of 2.50 g/cm^3^.

In terms of water absorption, natural aggregates demonstrated the lowest value at 1.0% (±0.1%), indicating minimal internal porosity. As the compositional complexity of aggregates increased, water absorption exhibited a stepwise rise: silica-rich: 3.2% (±0.3%); carbonaceous: 5.5% (±0.4%); alumina-rich: 8.0% (±0.6%). This trend clearly indicates that changes in aggregate composition significantly affect their moisture uptake behavior. Density measurements further reveal systematic differences in material performance. Natural aggregates achieved the highest values across all metrics—SSD density (2.65 ± 0.02 g/cm^3^), bulk density (2.62 ± 0.02 g/cm^3^), and apparent density (2.68 ± 0.02 g/cm^3^). In contrast, alumina-rich aggregates showed the lowest density values: an SSD density of 2.50 ± 0.05 g/cm^3^, a bulk density of 2.47 ± 0.05 g/cm^3^, and an apparent density of 2.53 ± 0.05 g/cm^3^. The silica-rich (2.60 ± 0.03 g/cm^3^) and carbonaceous (2.55 ± 0.04 g/cm^3^) aggregates exhibited intermediate characteristics, following a decreasing gradient of natural > silica-rich > carbonaceous > alumina-rich.

In addition, variability in the test data also followed a clear trend. For density measurements, the standard deviation increased from ±0.02 g/cm^3^ for natural aggregates to ±0.05 g/cm^3^ for alumina-rich aggregates. Similarly, the standard deviation of water absorption rose from ±0.1% to ±0.6%. This reflects the increasing heterogeneity and performance dispersion with higher degrees of weathering and mineralogical complexity.

### 3.2. Crushing Strength Performance

The crushing resistance of the natural and weathered aggregates (silica-rich, carbonaceous, and alumina-rich) was evaluated through systematic crushing value tests. The experimental results and fracture mode observations are summarized in Table 2.

The crushing value results clearly demonstrate the deterioration of aggregate integrity caused by weathering. Natural aggregates exhibited the best mechanical performance, with a crushing value of only 12.5% and minimal variation (±0.8%). Fracture patterns were dominated by angular breakage (90%), indicating a well-preserved mineral skeleton. In contrast, weathered aggregates displayed a progressive degradation trend: The silica-rich aggregate showed an increased crushing value of 17.5% (±1.8%), with a shift in fracture behavior toward granular disintegration (55%) and flake-like peeling (30%). The carbonaceous aggregate revealed a more severe degradation, with a crushing value of 23.8% (±2.2%). Flake peeling became the dominant mode (65%), accompanied by visible pulverization (20%). The alumina-rich aggregate demonstrated the weakest crushing resistance, reaching a value of 27.4% (±2.5%). Granular disintegration was predominant (70%), along with residual flake peeling (25%).

These findings indicate that increasing degrees of weathering not only reduce the mechanical integrity of aggregates but also alter their failure mechanisms—from intergranular fracture in natural aggregates to intra-structural disintegration in heavily weathered types. This transition provides macro-level evidence of microstructural deterioration.

### 3.3. Abrasion Resistance Performance

The abrasion resistance of the natural and weathered aggregates was evaluated using the Los Angeles abrasion test, following JTG E42-2005 standards [49]. The results are shown in Table 3.

As indicated in Table 3, the abrasion resistance performance of the four aggregate types exhibits a clear gradient. The natural aggregate showed the best resistance to abrasion, with the lowest LA abrasion value of 12.0%, and the smallest variability (±0.8%). In contrast, the weathered aggregates exhibited significantly higher abrasion values, indicating greater susceptibility to mechanical wear: silica-rich aggregate: 16.0% (±1.2%); carbonaceous aggregate: 22.0% (±1.8%); alumina-rich aggregate: 26.0% (±2.0%). The results reflect a performance degradation of 10.0–14.0 percentage points between natural and weathered aggregates, with the alumina-rich aggregate showing the poorest abrasion resistance and the highest data dispersion. Notably, the variability in test results also increased with the severity of weathering, especially in the carbonaceous and alumina-rich aggregates. This trend suggests that mineralogical heterogeneity and microstructural instability induced by weathering contribute to the observed mechanical deterioration and increased data fluctuation.

### 3.4. Microscopic Pore Structure Characteristics

**Figure 4:**
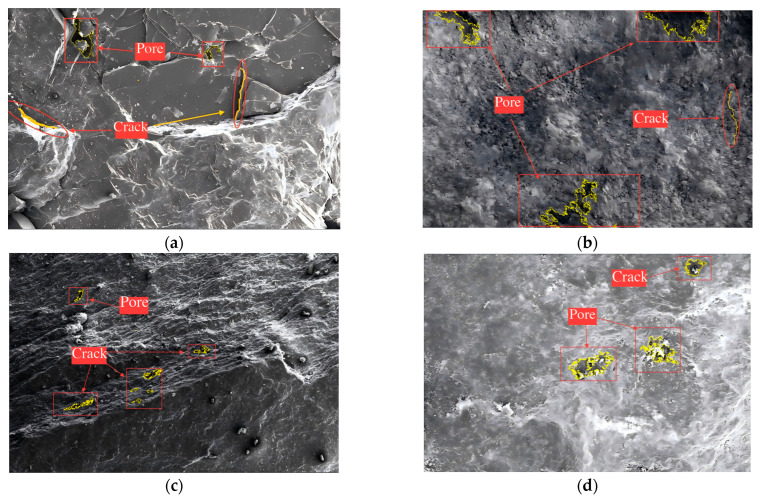
Surface micromorphology of natural and weathered aggregates under SEM, with labeled features highlighting pore structures and crack propagation. (**a**) Natural aggregate exhibiting a compact surface with isolated intergranular pores and incipient microcracks; (**b**) silica-rich aggregate displaying widespread pore formation and microcracks, reflecting degradation of the silicate matrix; (**c**) carbonaceous aggregate characterized by stratified flaky texture and weathering-induced fissures distributed along laminar surfaces; (**d**) alumina-rich aggregate showing extensive porous zones and fine cracks, indicating advanced chemical weathering and aluminum-enriched mineral disintegration. Pores and cracks are directly labeled on each image to enhance microstructural interpretation.

To further investigate the impact of weathering on the internal structure of aggregates, scanning electron microscopy (SEM) combined with ImageJ-based image analysis was applied to characterize the surface morphology and pore structure of natural, silica-rich, carbonaceous, and alumina-rich aggregates. The analysis focused on key indicators such as porosity, average pore size, microcrack density, and pore connectivity. Figure 4 presents representative SEM images at 5000× magnification for the four aggregate types, while Figure 4a–d shows the corresponding processed images for quantitative pore analysis. The microstructural parameter statistics are summarized in Table 4.

The natural aggregate shows a dense and compact surface, with clearly defined contours and sparse, isolated pores; the silica-rich aggregate displays granular detachment and localized pore etching, indicating incipient surface degradation; the carbonaceous aggregate exhibits irregular surface dissolution and distinct strip-like cracking; and the alumina-rich aggregate is the most structurally degraded, with numerous intersecting fissures and voids, reflecting severe weathering and structural disintegration. These morphological differences visually illustrate a progressive deterioration trend as weathering intensifies. The natural aggregate retains a well-preserved structure, while the weathered aggregates show increasing signs of surface fragmentation, crack propagation, and pore development. Such microstructural evolution is strongly linked to the decline in macroscopic mechanical performance.

**Figure 5:**
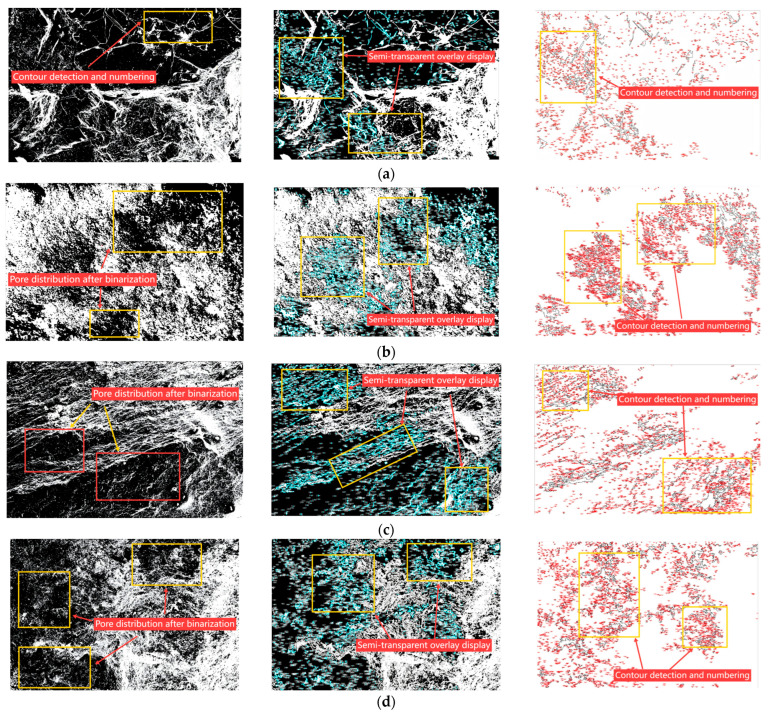
Image-based pore structure analysis of natural and weathered aggregates using SEM and ImageJ processing. (**a**) Natural aggregate: pore contours are identified through binarization, numbered, and visualized with semi-transparent overlays to highlight internal void distribution. (**b**) Silica-rich aggregate: evident pore clusters are detected post-binarization, with contour delineation illustrating pore morphology and connectivity. (**c**) Carbonaceous aggregate: laminar texture and stratified voids are highlighted via binary segmentation and overlay rendering. (**d**) Alumina-rich aggregate: dense and irregular pore networks are extracted and visualized, reflecting advanced disintegration during weathering. Each row displays, from left to right: the binarized image, semi-transparent overlay visualization, and contour detection with numbering for quantitative analysis.

Figure 5a–d illustrates the visualized pore structure of the four aggregates based on SEM images processed via ImageJ. The image analysis procedure included grayscale threshold segmentation, pore boundary recognition, connectivity assessment, and false-color mapping to highlight pore morphology and distribution. (Figure 5a) (natural aggregate): pores are scarce and mostly isolated, with no significant connectivity. (Figure 5b) (silica-rich aggregate): pore quantity increases with partial short-range connectivity. (Figure 5c) (carbonaceous aggregate): a large number of elongated microcracks are observed. (Figure 5d) (alumina-rich aggregate): a well-developed interconnected pore network is present, indicating severe structural deterioration.

Quantitative results for pore-related microstructural parameters are presented in Table 4.

The natural aggregate exhibited the lowest porosity (4.2%), smallest average pore size (0.12 μm), and lowest microcrack density (0.22 μm/μm^2^), along with a limited connected pore ratio (24.5%), indicating a compact and stable internal structure. In contrast, the alumina-rich aggregate had the most degraded microstructure: porosity reached 12.7%, average pore size expanded to 0.85 μm, microcrack density increased to 1.40 μm/μm^2^, and the connected pore ratio rose to 68.2%. This implies a high degree of structural continuity among voids, consistent with its poorest macroscopic performance.

Overall, the weathered aggregates displayed a clear trend of increasing porosity, widened pore size, greater crack density, and enhanced connectivity, indicating a transition toward more fragmented and permeable structures. This evolution explains the elevated water absorption and mechanical degradation observed at the macroscale. Therefore, in engineering practice, it is recommended to classify weathered aggregates based on these microstructural indicators. Dense, low-connectivity aggregates may be suitable for high-performance structural layers, while severely weathered materials with developed pore networks may require pretreatment (e.g., surface sealing or chemical modification) to enhance durability and reduce failure risk under extreme plateau conditions.

### 3.5. Elemental Distribution and Migration Behavior

**Figure 6:**
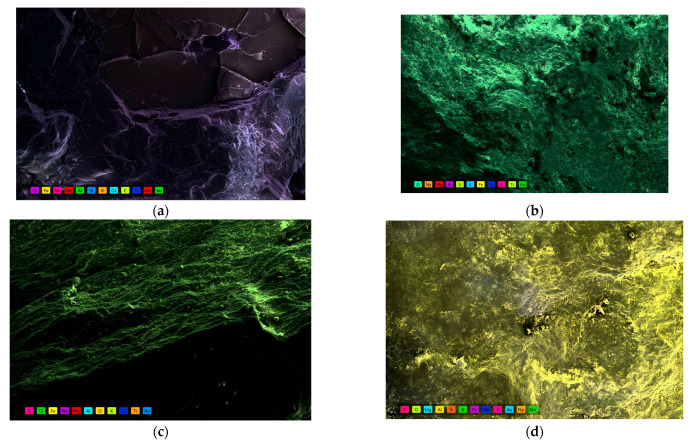
Elemental distribution maps of four aggregate types obtained via EDS area scans. (**a**) Natural aggregate shows homogeneous distribution of Si, Al, O, and minor Fe and Ca elements, reflecting a well-preserved silicate framework and limited elemental migration. (**b**) Silica-rich aggregate exhibits pronounced Si enrichment with reduced concentrations of Fe, C, and K, suggesting quartz retention and selective leaching of mobile ions. (**c**) Carbonaceous aggregate characterized by elevated C, Na, and Mg concentrations aligned along laminar microstructures, indicating early-stage clay mineral formation and organic–mineral association. (**d**) Alumina-rich aggregate displays a strong Al and Fe enrichment with concurrent Si depletion, implying advanced weathering, intense elemental migration, and the formation of secondary minerals such as hematite and illite.

To further investigate the effect of weathering on aggregate elemental composition and spatial migration behavior, SEM combined with energy-dispersive X-ray spectroscopy (EDS) was applied to analyze natural, silica-rich, carbonaceous, and alumina-rich aggregates. Both surface mapping and point-scan spectra were used to characterize elemental abundance, distribution uniformity, and compositional evolution across different weathering types. Figure 6 displays the EDS elemental maps for the four aggregate types. The natural aggregate exhibits a uniform distribution of major elements and an intact crystalline structure. The silica-rich aggregate shows pronounced Si enrichment zones. In contrast, the carbonaceous and alumina-rich aggregates display notable elemental concentrations near cracks and pores, suggesting active spatial migration and weathering-induced redistribution of elements.

**Figure 7:**
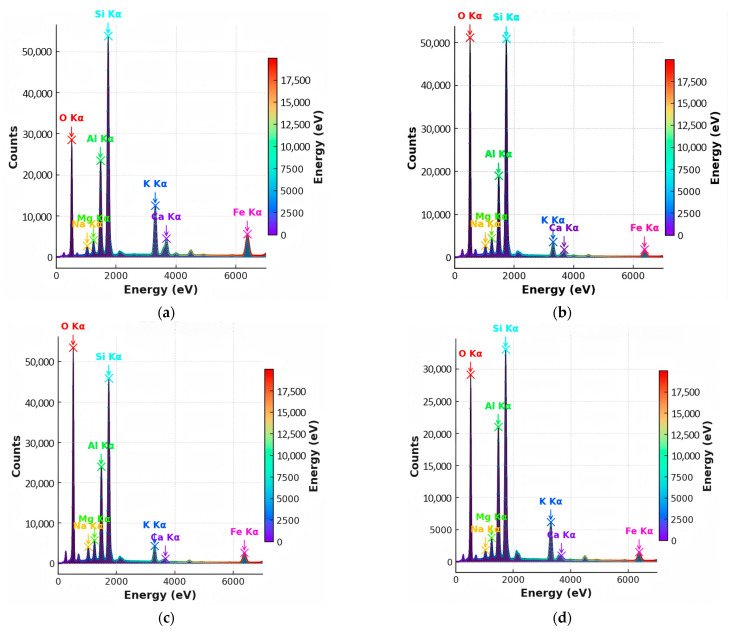
Energy-dispersive X-ray spectroscopy (EDS) spectra of four aggregate types. (**a**) Natural aggregate exhibits strong Si and O peaks, with moderate Al, Fe, Ca, and K signals, indicating a well-preserved silicate framework and minimal weathering. (**b**) Silica-rich aggregate shows pronounced Si enrichment and reduced presence of other elements, suggesting quartz retention and the leaching of soluble phases. (**c**) Carbonaceous aggregate displays relatively high levels of Na, Mg, and C, reflecting organic–mineral associations and early-stage clay mineral formation. (**d**) Alumina-rich aggregate characterized by elevated Al, Fe, and K and reduced Si, indicating advanced chemical weathering and transformation into secondary minerals such as illite and hematite. The spectra identify the elemental composition of each aggregate type and reveal key differences related to weathering.

Figure 7 compares the major element spectra among the four aggregate types, highlighting differences in peak intensity for Si, Al, C, Fe, and other constituents. In the natural aggregate, the dominant elements are Si, Al, C, and O. The Si/Al mass ratio is approximately 2.45, indicating a stable silicate-based mineral framework. The high carbon content (13.4 wt.%) may result from residual carbonates or organic matter. The silica-rich aggregate contains 33.8 wt.% Si, increasing the Si/Al ratio to 3.08, confirming significant quartz retention. Notably, C and Fe were undetected, suggesting that soluble phases have been leached during weathering. The carbonaceous aggregate retains 4.5 wt.% C and exhibits elevated Na (2.2 wt.%) and Mg (1.7 wt.%), indicating the presence of clay minerals such as montmorillonite and possibly organic–mineral complexes. Its Si/Al ratio drops to 2.20, reflecting structural changes. The alumina-rich aggregate, the most weathered sample, shows a substantial decrease in Si (21.1 wt.%) and an increase in Al (11.6 wt.%), resulting in a Si/Al ratio of 1.82. Significant enrichment of Fe (7.4 wt.%) and K (5.8 wt.%) was also observed. The absence of C, Ca, and Ti implies the transformation into secondary minerals, such as hematite and illite.

**Figure 8:**
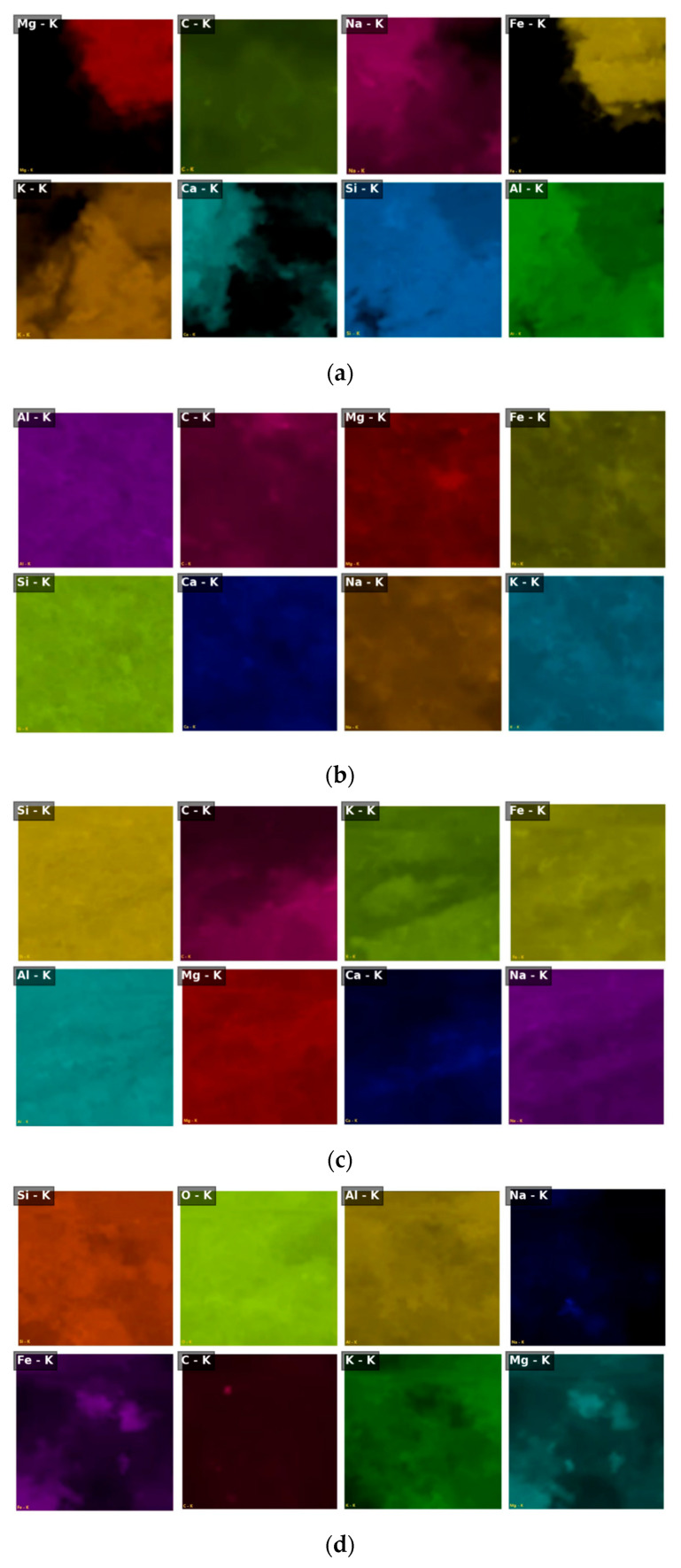
Elemental migration features in crack and pore regions of aggregates, based on EDS elemental mapping. (**a**) Natural aggregate: elemental distributions (e.g., Si, Al, Ca, Fe) remain uniform with no evident migration gradients across the matrix, indicating a chemically stable structure and negligible weathering-induced redistribution. (**b**) Silica-rich aggregate: Si shows relatively high concentration, while mobile ions such as Na, Ca, and K are partially leached, suggesting selective preservation of quartz and weak migration activity near microvoids. (**c**) Carbonaceous aggregate: C, Na, and Mg are enriched along microcrack regions, implying coupled migration of organic components and clay-forming cations; this supports early-stage montmorillonite development and organic–mineral associations. (**d**) Alumina-rich aggregate: a distinct “Si depletion–Fe and Al enrichment” pattern is observed near cracks and pore rims, accompanied by strong K and Mg accumulation. These trends reflect intense elemental reorganization, indicating transformation into secondary minerals such as illite and hematite.

These compositional trends reveal clear weathering pathways. With increasing weathering intensity, Si and C are progressively leached; Al, Fe, and K become enriched; and the mineral framework shifts from silicate dominance toward alumino-ferric clay and oxide minerals. Figure 8 presents localized EDS elemental distribution near cracks and pores, highlighting the migration behavior in weathered aggregates. In the alumina-rich aggregate, a pronounced “Si depletion–Fe enrichment” pattern is observed along crack boundaries. Compared to the matrix region, Si content near the crack decreases by 52%, while Fe increases by 218%, indicating a strong coupled migration mechanism and the potential formation of secondary iron-bearing minerals (e.g., hematite). In the carbonaceous aggregate, C and Mg tend to accumulate along microcracks. This suggests a co-migration of organic matter and clay-forming elements, possibly leading to the development of high-porosity, hydrophilic clay composites.

**Figure 9:**
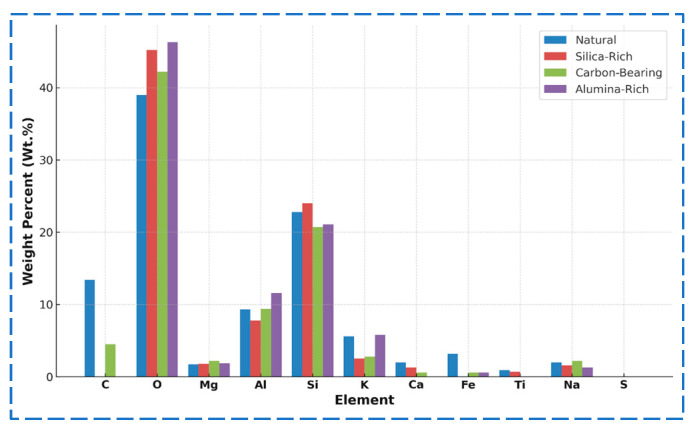
Elemental composition of aggregates (weight percent, wt.%).

**Figure 10:**
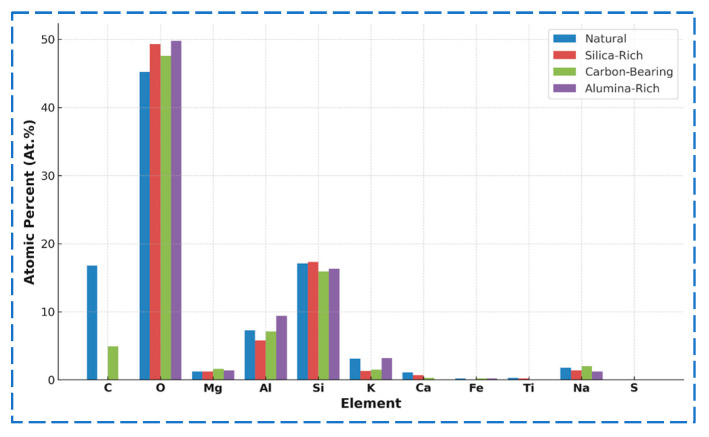
Elemental composition of aggregates (atomic percent, at.%).

To quantify the compositional evolution, the elemental contents were statistically analyzed in both weight percent (Wt.%) and atomic percent (At.%), as shown in Figure 9 and Figure 10, respectively. The natural aggregate features 29.1 wt.% Si and 11.9 wt.% Al (At.%: 22.5%, 9.2%), yielding a Si/Al ratio of 2.45, consistent with a stable silicate skeleton. In the alumina-rich aggregate, the most weathered sample, Si declines to 21.1 wt.%, while Al increases to 11.6 wt.%, resulting in a Si/Al ratio of 1.82. Notably, Fe and K are significantly enriched, supporting the presence of secondary iron–aluminum minerals and structural reconstitution. The silica-rich aggregate maintains the highest Si content (33.8 wt.%) and Si/Al ratio (3.08), although Na and K show considerable depletion, indicating selective preservation of quartz and leaching of alkali components. The carbonaceous aggregate sustains 4.5 wt.% C, along with relatively high levels of Na and Mg, reflecting a combined weathering mechanism involving organic–mineral interactions.

In summary, the four aggregate types show distinct patterns of elemental evolution: with increasing weathering, Si and C are gradually leached; Al, Fe, and K become concentrated, and the overall Si/Al ratio declines. This indicates a mineralogical transition from primary silicate structures to secondary clay and oxide phases—a key indicator of weathering intensity and structural transformation. These findings provide critical insight into the weathering-induced element migration pathways and their links to microstructural deterioration. Such compositional shifts explain the weakening of aggregate integrity and are foundational for developing a micro–macro coupling model, which is discussed in the next section.

### 3.6. Correlation Analysis Between Microstructure and Macroscopic Performance

To explore the intrinsic link between microstructural features and macroscopic performance of weathered aggregates from the Qinghai–Tibet Plateau, a gray entropy correlation analysis was conducted. This approach emphasizes the influence of two key micro-level parameters—Si/Al ratio and porosity—on major engineering performance indicators, including density, water absorption, crushing value, and abrasion resistance. Based on the experimental data presented in Table 1, Table 2, Table 3 and Table 4, a multiscale performance correlation model was established.

#### 3.6.1. Model Construction

**Figure 11:**
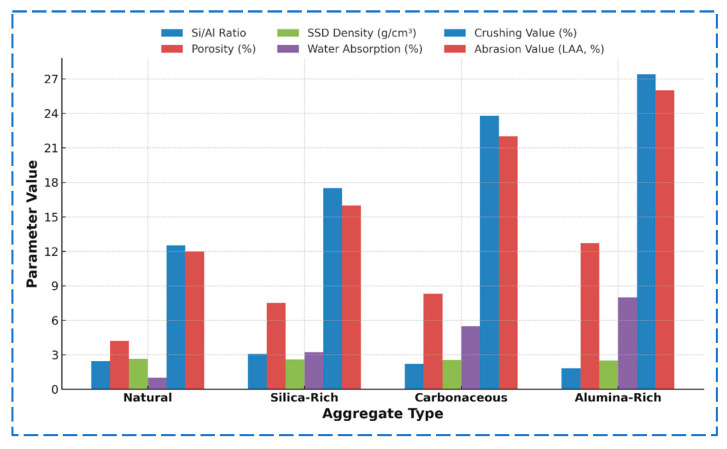
Microstructural and macroscopic performance data of aggregates.

A raw data matrix was constructed using measured microstructural and mechanical properties of the four aggregate types (natural, silica-rich, carbonaceous, alumina-rich), as shown in Figure 11. To eliminate dimensional inconsistency across different parameters, initial value normalization was applied using the following equation:

(8) $$x_{kj}^{\prime }=\frac{x_{kj}}{x_{k1}}$$

where $x_{kj}$ is the value of the j-th parameter for the k-th aggregate type; k = 1k = 1: natural aggregate; k = 2k = 2: silica-rich aggregate; k = 3k = 3: carbonaceous aggregate; k = 4k = 4: alumina-rich aggregate.

In this model, the Si/Al ratio $\left(X_{1}\right)$ and porosity $\left(X_{2}\right)$ are treated as comparative sequences, the macroscopic indicators $\left(Y_{j}\right)$ are treated as reference sequences, and the gray relational coefficient is calculated using the standard formula:

(9) $$\gamma_{ij}=\frac{\min+\zeta \cdot \max}{\left|x_{i}-y_{j}\right|+\zeta \cdot \max}$$

where min and max are the global minimum and maximum differences between the reference and comparative sequences, *ζ* is the resolution coefficient, typically set as 0.5 in engineering applications.

#### 3.6.2. Correlation Characteristics

Based on the gray entropy analysis, the relational degrees between the microstructural parameters (Si/Al ratio and porosity) and the macroscopic performance indicators were calculated. The results are shown in Table 5.

The results in Table 5 reveal the following key findings: The Si/Al ratio shows strong correlation with both SSD density (γ = 0.93) and crushing value (γ = 0.94), indicating its crucial role in determining structural stability and compressive strength. As weathering progresses and Si leaches out, the Si/Al ratio decreases, weakening the mineral skeleton and reducing mechanical integrity. For instance, in alumina-rich aggregates, the Si/Al ratio drops from 2.45 to 1.82, accompanied by a decline in SSD density from 2.65 to 2.50 g/cm^3^, and an increase in crushing value to 27.4%—a 119% rise compared to natural aggregates. The porosity is the dominant factor influencing water absorption (γ = 0.92) and abrasion resistance (γ = 0.91), reflecting its role in governing moisture sensitivity and durability. Increased porosity facilitates capillary infiltration and internal degradation. For example, the natural aggregate has a porosity of 4.2% and a water absorption of 1.0%, while the alumina-rich aggregate shows 12.7% porosity and 8.0% water absorption—a 700% increase. The abrasion value similarly rises from 12.0% to 26.0%. The silica-rich aggregate, despite retaining a high Si/Al ratio (3.08), exhibits moderate degradation due to elevated porosity (7.5%), resulting in increased abrasion (16.0%). The carbonaceous and alumina-rich aggregates suffer simultaneous effects of decreased Si/Al ratio and increased porosity, leading to more severe performance deterioration across all indicators.

In summary, the Si/Al ratio primarily governs mechanical strength and density, while porosity controls moisture sensitivity and wear resistance. These two microstructural parameters jointly determine the engineering behavior of weathered aggregates, forming the basis of a dual-factor evaluation framework that supports classification and treatment strategies for aggregate applications in high-altitude regions.

## 4. Conclusions

In this study, a multiscale analytical framework was developed to assess the engineering performance of weathered aggregates from representative regions of the Qinghai–Tibet Plateau. The framework integrated macro-scale mechanical tests, microstructural characterization, and chemical composition analysis to reveal the degradation mechanisms and controlling factors associated with weathering. A dual-factor control model based on the Si/Al ratio and porosity was proposed, and a gray entropy correlation model was constructed to bridge microstructure and macroscopic performance. The main conclusions are as follows:

(1) Weathering significantly affects the physical and mechanical properties of aggregates. Natural aggregates exhibited high compactness, low water absorption, and strong resistance to crushing and abrasion. In contrast, weathered aggregates showed increased water absorption, reduced density, and deteriorated strength and wear resistance. Among them, alumina-rich aggregates experienced the most severe degradation, with crushing and abrasion values more than twice those of the natural aggregates, indicating substantial loss of structural integrity and load-bearing capacity.
(2) Distinct microstructural patterns are observed among different weathering types. Natural aggregates had the lowest porosity, the smallest average pore size, and the lowest microcrack density, reflecting a dense internal structure. With increasing weathering intensity, silica-rich, carbonaceous, and alumina-rich aggregates showed synchronous increases in porosity parameters. Notably, the alumina-rich aggregate had a porosity of 12.7%, an average pore size of 0.85 μm, and a connected pore ratio of 68.2%, indicating a high degree of fragmentation and structural continuity.
(3) Elemental migration features clarify the weathering mechanisms. Natural aggregates were dominated by stable Si–Al frameworks. The silica-rich aggregate showed Si enrichment, consistent with quartz preservation. The carbonaceous aggregate was rich in C, Na, and Mg, indicating organic accumulation and clay mineral evolution. In the alumina-rich aggregate, Si was strongly depleted while Al, Fe, and K were enriched, representing a typical “Si-leaching–Al/Fe-enrichment” weathering pathway, and the formation of secondary minerals such as hematite and illite.
(4) Si/Al ratio and porosity serve as dominant microstructural control factors with strong correlations to macroscopic performance. Gray entropy analysis showed that the Si/Al ratio primarily influences density and compressive resistance, while porosity governs water absorption and abrasion durability. The correlation between the Si/Al ratio and crushing value reached 0.94, while the correlation between porosity and water absorption reached 0.92, confirming the coupled mechanism of “skeleton integrity—structural permeability” in governing aggregate performance.
(5) The proposed dual-factor evaluation model based on Si/Al ratio and porosity enables effective classification and engineering application of weathered aggregates. Aggregates located in the first quadrant of the Si/Al–porosity plot exhibit superior structure and are suitable for use in high-grade structural layers. Aggregates in the third quadrant, characterized by high porosity and low skeletal integrity, require caution and should be modified (e.g., sealed or stabilized) before use in sub-layers.

In conclusion, a systematic coupling relationship exists between the multiscale structural features and performance of weathered aggregates from the Qinghai–Tibet Plateau. The dual-factor control framework and gray entropy correlation model proposed in this study provide a scientific basis for material selection, durability design, and classification strategies under extreme plateau conditions. Future research should focus on modification and strengthening technologies for weathered aggregates. Considering their high porosity, strong water absorption, and weak structural cohesion, treatment methods such as surface sealing with silane coupling agents should be further explored to enhance hydrophobicity and resistance to moisture-induced damage.

## 5. Discussion

### 5.1. Integrated Interpretation and Scientific Positioning

This study provides a multiscale investigation into the degradation behavior of weathered aggregates from the Qinghai–Tibet Plateau, establishing a coupled relationship between microstructural indicators and macroscopic engineering performance. The observed deterioration—reflected by increasing porosity, pore connectivity, and elemental redistribution—is consistent with the chemical weathering theories proposed in recent high-altitude material science literature [1,9,21]. However, unlike previous studies that rely primarily on mineralogical classification or qualitative SEM imaging, our approach quantitatively links two key indicators—Si/Al ratio and porosity—to strength and durability metrics through a gray entropy-based correlation model.

### 5.2. Comparative Analysis with Existing Literature

To demonstrate the novelty and reliability of the proposed framework, Table 6 compares our results with those reported for recycled, weathered, and treated aggregates across varied environments. The alumina-rich aggregate from this study exhibits a porosity of 12.7%, a crushing value of 27.4%, and an abrasion loss of 26.0%—substantially higher than recycled concrete aggregates (typically 6–10% porosity, <22% crushing value) [19,33,40]. In contrast to traditional evaluation models (e.g., ITZ indices, water absorption ratio, or empirical regression), the dual-factor Si/Al–porosity system exhibits enhanced sensitivity to degradation intensity and better performance classification accuracy under complex field conditions [11,14,39].

### 5.3. Engineering Application and Generalization Potential

The dual-factor framework demonstrates strong practicality. Aggregates with a Si/Al ratio > 2.5 and porosity < 6% exhibit structural reliability, making them suitable for surface layers or load-bearing base courses in cold-region pavements. Conversely, aggregates with Si/Al < 2.0 and porosity > 10% require pretreatment—such as silane sealing or nano-silica impregnation—before incorporation into subgrades [14,38,41]. This classification strategy supports intelligent material selection and quality control across high-altitude engineering projects in Asia, Europe, and South America, especially in freeze–thaw-sensitive or sulfate-rich zones [8,10,37].

### 5.4. Limitations and Future Perspectives (Revised for Seamless Integration)

As discussed in the previous sections, the dual-factor framework based on Si/Al ratio and porosity offers a quantifiable and degradation-sensitive classification approach for weathered aggregates, outperforming traditional mineral-based or empirical models. However, despite its demonstrated effectiveness, several limitations constrain its broader applicability. The Si/Al ratio, while a useful proxy, may oversimplify the underlying multicomponent mineralogical transformations, particularly involving Fe-, Mg-, and alkali-bearing phases, which play crucial roles in weathering-induced phase evolution. Additionally, the current study is limited to granite-derived aggregates sourced from the eastern Qinghai–Tibet Plateau, where high-altitude conditions (elevation > 3500 m), frequent freeze–thaw cycles (>60/year), and intense UV radiation dominate the degradation mechanisms. As such, the model’s transferability to tropical, coastal, or marine climates remains unverified. Further cross-lithological validation and regional adaptation are needed to enhance its generalization. Moreover, the framework remains static and lacks coupling with time-dependent deterioration models or reactive transport simulations that are critical for durability forecasting under cyclic environmental loads. Future work should incorporate machine learning algorithms trained on microstructural–mechanical datasets, digital pore network modeling, and accelerated weathering experiments (e.g., sulfate attack, moisture-driven cracking, UV degradation) to capture dynamic deterioration trends. In parallel, the development of scalable reinforcement techniques—such as nanoparticle sealing, reactive silicate impregnation, and bio-mineralization—will be essential to enhance the long-term reliability of high-porosity, low-strength aggregates in climate-sensitive infrastructure applications.

## Figures and Tables

**Table 1:** Density and water absorption of aggregates.

Aggregate Type	Water Absorption (%)	SSD Density (g/cm^3^)	Bulk Density (g/cm^3^)	Apparent Density (g/cm^3^)
Natural Aggregate	1.0 ± 0.1	2.65 ± 0.02	2.62 ± 0.02	2.68 ± 0.02
Silica-Rich Aggregate	3.2 ± 0.3	2.60 ± 0.03	2.58 ± 0.03	2.63 ± 0.03
Carbonaceous Aggregate	5.5 ± 0.4	2.55 ± 0.04	2.52 ± 0.04	2.58 ± 0.04
Alumina-Rich Aggregate	8.0 ± 0.6	2.50 ± 0.05	2.47 ± 0.05	2.53 ± 0.05

**Table 2:** Crushing value test results of aggregates.

Aggregate Type	Crushing Value (%)	Deviation Range	Dominant Fracture Mode (Proportion)
Natural Aggregate	12.5	±0.8	Angular fracture (90%)
Silica-Rich Aggregate	17.5	±1.8	Granular disintegration (55%) + Flake peeling (30%)
Carbonaceous Aggregate	23.8	±2.2	Flake peeling (65%) + Pulverization (20%)
Alumina-Rich Aggregate	27.4	±2.5	Granular disintegration (70%) + Flake peeling (25%)

**Table 3:** Los Angeles abrasion value of aggregates.

Aggregate Type	LA Abrasion Value (%)	Deviation Range
Natural Aggregate	12.0	±0.8
Silica-Rich Aggregate	16.0	±1.2
Carbonaceous Aggregate	22.0	±1.8
Alumina-Rich Aggregate	26.0	±2.0

**Table 4:** Microscopic pore structure parameters of aggregates.

Aggregate Type	Porosity (%)	Avg. Pore Size (μm)	Microcrack Density (μm/μm^2^)	Connected Pore Ratio (%)
Natural Aggregate	4.2 ± 0.3	0.12 ± 0.02	0.22 ± 0.04	24.5 ± 2.8
Silica-Rich Aggregate	7.5 ± 0.6	0.25 ± 0.05	0.58 ± 0.10	42.6 ± 3.5
Carbonaceous Aggregate	8.3 ± 0.7	0.48 ± 0.08	0.82 ± 0.15	52.8 ± 4.1
Alumina-Rich Aggregate	12.7 ± 1.0	0.85 ± 0.16	1.40 ± 0.22	68.2 ± 5.0

**Table 5:** Gray relational degree between microstructure and performance indicators.

Macroscopic Performance Indicator	Relational Degree with Si/Al (γ_1j_)	Relational Degree with Porosity (γ_2j_)	Dominant Factor
SSD Density	0.93	0.85	Si/Al Ratio
Water Absorption	0.81	0.92	Porosity
Crushing Value	0.94	0.89	Si/Al Ratio
Abrasion Value	0.83	0.91	Porosity

**Table 6:** Comparison of aggregate properties and evaluation frameworks in existing studies and this work.

Study/Source	Aggregate Type	Porosity (%)	Si/Al Ratio	Crushing Value (%)	Abrasion Loss (%)
This Study	Alumina-Rich Weathered Aggregate	12.7	1.82	27.4	26.0
Liu et al. (2023) [34]	Recycled Concrete Aggregate	8.4	–	20.1	18.3
Buking et al. (2023) [6]	Weathered Granite	7.2	2.01	21.5	20.8
Zhao et al. (2021) [10]	Reclaimed Asphalt Aggregate	9.5	–	22.3	17.6
Gong et al. (2023) [33]	Porous Cement-Based Aggregate	10.3	2.04	23.2	19.1
Zou et al. (2022) [30]	Nano-MgO Magnesia Aggregate	6.0	2.42	18.5	14.8
Tran et al. (2024) [37]	Pervious Concrete with Recycled Aggregate	11.1	–	24.6	21.3

## Data Availability

The original contributions presented in this study are included in the article material. Further inquiries can be directed to the first author.
